# Molecular and Immunological Identification of Low Allergenic Fruits among Old and New Apple Varieties

**DOI:** 10.3390/ijms22073527

**Published:** 2021-03-29

**Authors:** Aleksandra Siekierzynska, Dorota Piasecka-Kwiatkowska, Wojciech Litwinczuk, Marta Burzynska, Aleksander Myszka, Pawel Karpinski, Elzbieta Zygala, Narcyz Piorecki, Ewa Springer, Tomasz Sozanski

**Affiliations:** 1 Department of Physiology and Plant Biotechnology, Institute of Agricultural Sciences, Land Management and Environmental Protection, University of Rzeszow, Cwiklinskiej 2, 35-601 Rzeszow, Poland; wlitw@ur.edu.pl; 2 Department of Food Biochemistry and Analysis, Poznan University of Life Sciences, Mazowiecka 48, 60-623 Poznan, Poland; dorota.piasecka-kwiatkowska@up.poznan.pl (D.P.-K.); marta.burzynska@up.poznan.pl (M.B.); 3 Institute of Medical Sciences, University of Rzeszow, Rejtana 16 c, 35-959 Rzeszow, Poland; amyszka@ur.edu.pl; 4 Department of Genetics, Wroclaw Medical University, Marcinkowskiego 1, 50-368 Wroclaw, Poland; polemiraza@poczta.fm; 5 Laboratory of Genomics & Bioinformatics, Institute of Immunology and Experimental Therapy, Polish Academy of Sciences, 53-114 Wroclaw, Poland; 6 Arboretum and Department of Physiography in Bolestraszyce, 37-700 Przemysl, Poland; e.zygala@wp.pl (E.Z.); arboretum@poczta.onet.pl or; 7 Department of Human Sciences, Institute of Physical Culture Sciences, University of Rzeszow, Towarnickiego 3, 35-959 Rzeszow, Poland; 8 Center for Allergy Diagnostics and Treatment SNZOZ Alergologia Plus, 60-693 Poznan, Poland; alergologiaplus@wp.pl; 9 Department of Pharmacology, Wroclaw Medical University, Jana Mikulicza-Radeckiego 2, 50-345 Wroclaw, Poland; tsoz@wp.pl

**Keywords:** apple allergy, Mal d 1, gene expression, immunoreactivity, old apple varieties, hypoallergenic

## Abstract

About 50–70% of patients allergic to birch pollen suffer from sensitization after apple ingestion. Apple allergenicity was established in only few varieties. Studies were performed on apple fruits of 21 traditional and nine modern varieties organically, intensively, or integratively produced. The aim of the study was to assess whether the factors like cultivation method, maturity stage, genotype, or type of tissue place an impact on the allergenic potential of apples. To answer these questions, we used semiquantitative real-time PCR, ELISA, and immunoblotting. Apple allergen genes present divergent expression across apple cultivars. Expression of the *Mal d 1.06A* correlates with the Mal d 1 level and is affected by the cultivation method and maturity of the fruit. The content of the main allergen Mal d 1 varied widely across cultivars. Interestingly, in our study, the Gala variety presented a low Mal d 1 concentration regardless of the cultivation method. Based on the *Mal d 1.06A* expression, the Mal d 1 protein content, and the immunoreactivity assay, the Kandil Sinap, Kosztela, Rumianka from Alma-Ata, Kantówka Gdańska, Reinette Coulon, and Gala cultivars emerged as potentially hypoallergenic apple cultivars. Our study allowed distinguishing between potentially low, medium, and highly allergenic varieties.

## 1. Introduction

About 5–8% of children and 2–3% of adults suffer from a food allergy [1]. In Northern and Central Europe, sensitization to apples is associated with the cross-reactive birch pollen aeroallergen due to the structural homology of allergenic proteins. About 50–70% of pollen-sensitized patients suffer from oral allergy syndrome (OAS) after fresh apple ingestion [2].

So far, in apples (*Malus domestica* Borkh.), four clinically relevant allergens have been identified: Mal d 1, Mal d 2, Mal d 3, and Mal d 4 [3]. In Northern Europe, Mal d 1 is the major allergen that causes allergic reactions to fruit. Mal d 1 is very similar to the main birch pollen allergen Bet v 1 and has similar epitopes for IgE antibodies, resulting in cross-reactions [4].

Two other proteins, Mal d 2 and Mal d 4 of less clinical importance, are also associated with hyperreactivity to apples. In Mediterranean countries, allergic reactions are mainly caused by the Mal d 3 protein [5,6,7].

The content of allergens and/or the expression of genes encoding the main apple fruit allergenic proteins has been characterized in only few varieties. Currently, only cv. Santana, cv. Elise, and cv. Topaz are considered to be less allergenic and well-tolerated by allergic patients [8,9,10]. The previous findings showed a relationship between the risk of allergic reaction with the variant (genotype), degree of maturity and storage behavior, mainly concerning the *Mal d 1* gene expression or the protein level. These analyses were carried out only on the Golden Delicious, Granny Smith, Fuji, Santana, Cox’s Orange Pippin, Topaz, and Braeburn cultivars [7]. There is an interesting question of whether other apple tree cultivars, traditional/old and new ones (bred before and after the “Green Revolution”, respectively), can have a low allergic potential. The issue of the expression of genes encoding all four main allergens in apples is poorly understood. There is also insufficient information about the influence of the cultivation method, maturity degree, and storage time, and only in the abovementioned apple cultivars.

The present study aimed to assess the level of the expression of genes encoding four main apple allergens and also the level of the Mal d 1 protein in 21 traditional/old and nine modern/new apple fruit varieties. We hypothesized that the factors like cultivation method, maturity stage, genotype, or type of tissue place an impact on allergenicity. To answer these questions, we used semiquantitative real-time PCR, ELISA, and immunoblotting techniques.

## 2. Results

The Results section shows the average expression of the analyzed genes, the content of the Mal d 1 protein, and the results of a serum immunoreactivity assay. The supplementary data show the gene expression in individual cultivars. Clustering analysis is presented in the Discussion.

### 2.1. Mal d 1

We investigated the expression of two isoforms of the *Mal d 1* gene (*Mal d 1.06A* and *Mal d 1.01*) and Mal d 1 protein concentration in all of the analyzed cultivars (Table 1 and Table 2, Figures S1–S4).

### 2.2. Mal d 1.06A

We observed that the gene expression of *Mal d 1.06A* is higher in old apple cultivars than in new ones (*p* = 0.000143). The expression of *Mal d 1.06A* was higher in the skin than in the flesh in old varieties (*p* = 0.053). The cultivation method did not influence the expression. In cv. Golden Delicious, cv. Idared, cv. Jonagored, cv. Gold Millennium, and cv. Gala, the expression of *Mal d 1.06A* positively correlated with the fruit maturity (Pearson’s r coeff. = 0.52, *p* = 0.038, Table 3). The expression of *Mal d 1.06A* positively correlated with the immunoreactivity of patients’ sera (Table 4).

### 2.3. Mal d 1.01

We observed that the gene expression of *Mal d 1.01* significantly differs between old and new apple cultivars (*p* = 0.013). The level of the *Mal d 1.01* transcript was higher in organically farmed apples than in the apples cultivated using the integrated method (*p* = 0.01) (Figure S4). The expression of *Mal d 1.01* was higher in the skin than in the flesh in old varieties (*p* = 0.00001). Our study revealed that in cv. Golden Delicious, cv. Idared, cv. Jonagored, cv. Gold Millennium, and cv. Gala, the expression of *Mal d 1.01* positively correlated with the fruit maturity (Pearson’s r coeff. = 0.61, *p* = 0.012, Table 3). We observed a positive correlation of the *Mal d 1.01* expression with immunoreactivity (Pearson’s r coeff. = 0.3851, *p* = 0.036).

### 2.4. Mal d 1.06A vs. Mal d 1.01

Among old varieties, the level of *Mal d 1.06A* expression was lower than of *Mal d 1.01* in the average expression and in the skin (*p* = 0.008 and *p* = 0.018, respectively) (Table 1).

### 2.5. Mal d 1 Protein

The content of the Mal d 1 protein was assessed in the apple flesh among old organic, new organic, and new-intensively cultivated apple trees (Table 2). Differences in medians between the analyzed groups were not statistically significant. We revealed that *Mal d 1.06A* expression correlates with the Mal d 1 protein content (Pearson’s r coefficient = 0.38, *p* = 0.036) (Table 3). Nonetheless, *Mal d 1.01* expression did not correlate with the main allergen content. We observed a positive correlation between the Mal d 1 content and immunoreactivity (r = 0.37, *p* = 0.04) (Table 4).

### 2.6. Mal d 2.01

The average expression in the group of old varieties differs significantly from all new and organic new cultivars (*p* = 0.043 and *p* = 0.025, respectively) (Table 1, Figure S5). The method of cultivation influences the *Mal d 2.01* gene expression (*p* < 0.04). The organic method, contrary to the intensive method, decreased the level of average expression and expression in the flesh (*p* < 0.05) (Table 1, Figure S6).

The expression of *Mal d 2.01* did not correlate with fruit maturity and immunoreactivity of patients’ sera.

### 2.7. Mal d 3.01

Old and new cultivars did not differ with regard to the *Mal d 3.01* transcript level. Across cultivars, we found that apple skin contains about two times more *Mal d 3.01* transcripts than the flesh (*p* < 0.05). Among old cultivars, only in cv. Oberland Raspberry Apple, cv. Kandil Sinap, and cv. Grochówka, the gene expression was raised in the flesh as much as in the skin (Figure S7). The organic and intensive methods, contrary to the integrated method, significantly enhance the expression in the skin (*p* < 0.05) (Figure S8). There was no correlation between expression, maturity status, and immunoreactivity of patients’ sera.

### 2.8. Mal d 4.01

New cultivars are characterized by the similar expression level both in the skin and flesh (Pearson’s r coeff. = 0.71, *p* < 0.01) (Figures S9 and S10). The method of cultivation did not influence the Mal d 4.1 expression. The maturity level positively correlated with the expression of Mal d 4.01 (r = 0.62, *p* = 0.011). There was no correlation with immunoreactivity.

### 2.9. Hierarchical Classification on Principal Components (HCPC)

**Figure 1:**
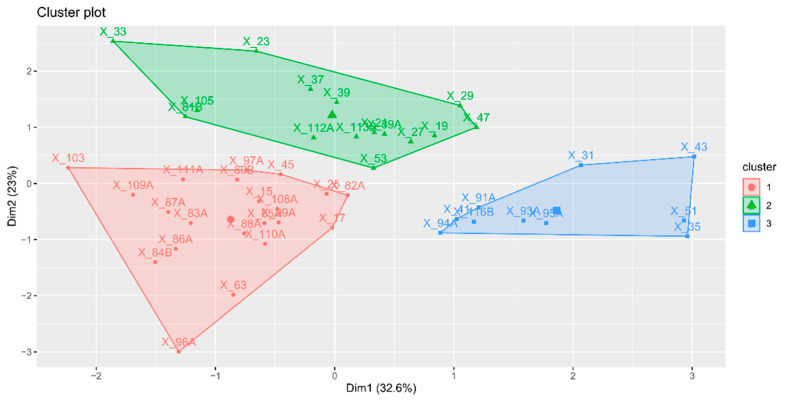
Hierarchical classification on principal components (HCPC) based on the gene expression of *Mal d 1.01*, *Mal d 1.06A*, *Mal d 2.01*, *Mal d 3.01*, and *Mal d 4.01*, in the flesh. Sample names are listed in Table 5.

The gene expression data (assessed in the fruit flesh) were integrated by means of principal component analysis to give an overview how the different cultivars behave in terms of all of the measured parameters. The first two components explained 55.6% of the total variance. The first principal component was able to explain 32.6% of the total variance observed in the analysis. The second component explained 23% of the total variance and led us to distinguish cultivar groups of low (in green), medium (in red), and high expression (in blue). Most of the old varieties display higher variability than the new ones (Figure 1).

## 3. Discussion

Our goal was to determine if apple allergenicity relies on factors like genotype, tissue type, cultivation method, maturity stage, and patients’ sera immunoreactivity. We showed that some of the old cultivars like Kandil Sinap, Ruminaka from Alma-Ata, Kantówka Gdańska, and Reinette Coulon, and new ones (Gala) can be hypoallergenic. By involving gene expression analysis, we could show that divergent isoforms of the *Mal d 1* gene could have a different impact on apple allergenicity.

Patients sensitized to apples report the severity of their symptoms depends on the variety and fruit maturity [11]. Mal d 1 is heat-labile and susceptible to digestion and symptoms are mainly connected with OAS, rarely with the gastrointestinal tract. Variability in the allergic potency might result from the different expression level of the Mal d isoforms. In our study, we determined the factors affecting the expression of genes encoding four main apple allergens.

*Mal d 1* presents divergent expression across apple cultivars. The expression of *Mal d 1.06A* and *Mal d 1.01* was lower in apple flesh contrary to apple skin, similarly to the study of Pagliarani et al. [12]. Furthermore, Schmitz–Eibereger et al. revealed a higher content of the main apple allergen Mal d 1 in the skin than in the flesh [13]. Elevated expression in the skin can be due to the protein function connected with fungal and bacterial infection response [14]. We indicated that the expression of *Mal d 1.06A*, contrary to *Mal d 1.01*, correlates with the Mal d 1 protein level (Pearson’s r coeff. = 0.38, *p* = 0.036) (Table 1); this indication is consistent with the results of other authors [5,7]. Moreover, we observed that *Mal d 1.01* expression was higher than of *Mal d 1.06A*, similar to the findings of Yang et al. [15].

According to our research, the cultivation method significantly influences the expression of *Mal d 1.01*. This is the first report revealing that organically and intensively farmed apples have a significantly elevated expression of *Mal d 1.01* compared to the integratively farmed ones (Table 1). It suggests that the *Mal d 1.01* isoform can be involved in response to the anti-pathogenic reaction and pesticide treatment as well. This hypothesis is consistent with the findings of Matthes and Schmitz–Eiberger el al. [9] who showed that pesticide treatment lead to an even more robust response than any biotic factors. What is more, Beuning [16] showed an elevated level of PR (pathogenesis-related proteins, including Mal d 1) during ripening, disease infection, and in response to environmental factors.

We observed that *Mal d 1.06A* is significantly correlated with immunoreactivity of patients’ sera, opposite to *Mal d 1.01*; this suggests that the *Mal d 1.06A* isoform has a major impact on apple allergenicity.

Our study also revealed the expression of *Mal d 1.06A* and *Mal d 1.01* positively correlates with fruit maturity (Pearson’s r coeff. = 0.52, *p* = 0.038; Pearson’s r coeff. = 0.61, *p* = 0.012, respectively, Table 3); this is consistent with the findings of Schmitz-Eiberger and Matthes [13].

Molecular studies of another apple allergen Mal d 2 are very few up to date. In our study, old apple cultivars had a higher transcription level of the *Mal d 2.01* isoform than new cultivars (*p* = 0.043). The method of cultivation influences the *Mal d 2.01* gene expression, e.g., the organic method decreased the transcript level in the flesh (*p* < 0.05). These findings indicate that genotype and farming method have an impact on the *Mal d 2.01* expression. Differences in the expression can be connected with the plant–pathogen reaction due to the Mal d 2 protein belonging to class PR-5. Gau et al. [17] showed that a high protein content of Mal d 2 was detected in scab-resistant cultivar Remo, suggesting a protective role against pathogens. Moreover, in a susceptible cultivar Elstar, after inoculation of pathogens, the concentration of the Mal d 2 protein increased [17].

Hsieh et al. [18] identified Mal d 2 as an in vitro reactive allergen among 75% of apple allergic patients in the USA; in turn, we did not observe the correlation of the gene expression with immunoreactivity, which may result from different etiology of sensitization to Mal d 1 and Mal d 2.

We established that the cultivation method affected the *Mal d 3.01* gene expression in the skin, but not in the flesh, in the group of new apple cultivars. Those findings are consistent with the Borges group [19], who proved the stable level of the *Mal d 3.01* gene in the flesh and the accumulation of the transcript in the skin. The biological role of the protein is participation in the cutin synthesis, and it can be accumulated in the epidermal layer of a plant [20]. This could explain why the expression of the *Mal d 3.01* gene isoform in the skin was elevated compared to apple flesh. Taking into account environmental factors affecting the expression of PR proteins, Gau et al. [17] showed that Mal d 3 is down-regulated during the pathogen infection. In turn, we observed the enhanced expression in the apple skin in organic and intensive methods (Table 1).

Mal d 3 allergen mostly sensitizes patients with a previous allergy to peaches, mainly in the Mediterranean. Unlike the allergy to Mal d 1, the symptoms whereof are predominantly limited to OAS, sensitization to Mal d 3 has serious, even life-threatening consequences. Mal d 3 is one of the non-specific lipid transfer proteins resistant to temperature or digestive enzymes, which is believed to be the factor causing reactions from mild to severe after ingestion [5]. It is worth noting that Fernandez–Rivas et al. [21] found that birch pollen sensitization was associated with 3.5-times decreased risk of Mal d 3 sensitization, whereas the allergy to mugwort and plane trees increased this risk 2.3–2.8-fold, respectively. The results of our study showed that *Mal d 3* expression does not correlate with immunoreactivity of sera of the patients sensitized to birch pollen, which is consistent with the above findings.

The last of the main analyzed allergens in apples was Mal d 4, profilin. We observed a stable expression level across the analyzed cultivars with a similar level in the flesh and skin, which might be a consequence of the Mal d 4’s biological function. Profilins are probably involved in signal transduction cascades and cytoskeleton organization, covering essential cellular functions [22], with a constitutive expression similar to actin [15]. We assumed the positive correlation of *Mal d 4.01* expression with fruit maturity (Pearson’s r’s coefficient = 0.62, *p* = 0.011); however, in case of prolonged storage, the Mal d 4 gene expression can be downregulated as shown by Botton et al. [7]. Mal d 4 causes rather mild symptoms in allergic patients, mostly OAS [23]. Profilins are quite sensitive to heat denaturation and gastric digestion, and thus food allergies caused by profilin are usually confined to the oral allergy syndrome elicited by fresh apple consumption. Sensitization to Mal d 4 occurs in Northern and Central Europe, similar to Mal d 1. Nevertheless, in the SAFE study, the prevalence of IgE specific to profilin was higher in Southern Europe patients than in the Central and Northern Europe patients [24]. Moreover, sera from patients with pollen allergy sensitized to profilin commonly show IgE cross-reactivity to fruits and vegetables. In the birch–Rosaceae fruit and the birch–mugwort–celery–spice cross-reactivity, profilin can play the role of the sensitizing agent [24]. However, in our studies, *Mal d 4.01* expression did not correlate with immunoreactivity of birch pollen-sensitized patients’ sera, what could stem from different sensitizing factor.

Assessing the simultaneous expression of four main allergen genes across all the studied cultivars, we used hierarchical classification on principal components (HCPC) and we revealed three clusters, the cluster in green covering low-expressed, the cluster in blue covering highly expressed, and the cluster in red covering medium-expressed apple allergen genes (Figure 1). We noticed that most of the old varieties display higher variability in gene expression than the new ones. What is more, the Mal d 1 protein content also varied widely across cultivars (from 0.3 up to 37.7 µg/g FW), showing a wide range of biodiversity (coefficient of variation—99.6% and 68.6% in old and new varieties, respectively). The large variation in respect to the content of Mal d 1 in the old varieties compared to the new ones indicates old varieties as a rich source of potentially low allergenic apples (Table 2).

**Figure 2:**
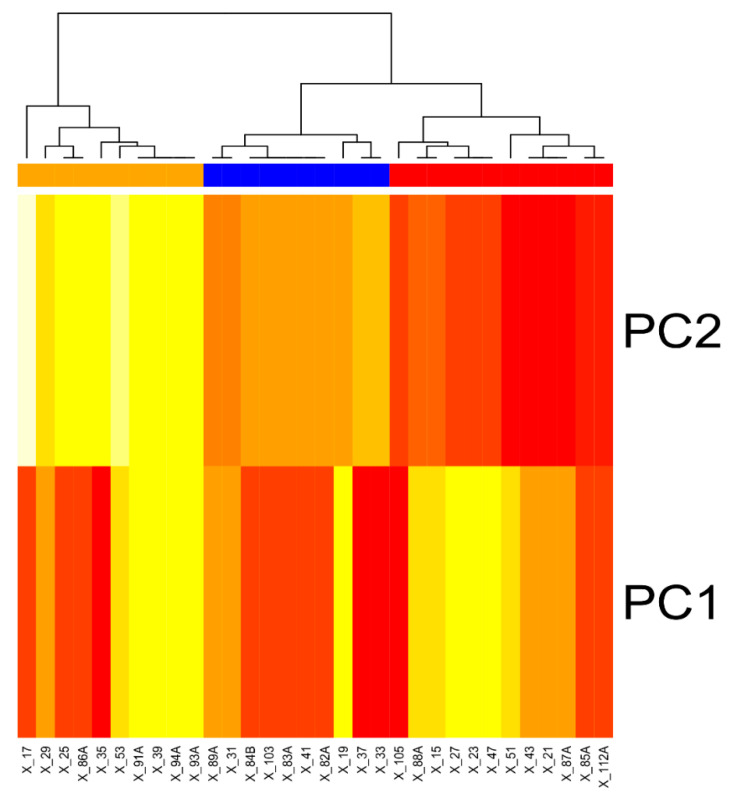
Unsupervised integrative clustering (UIC) based on the expression of *Mal d 1.06A*, Mal d 1 protein concentration, and immunoreactivity of patients’ sera with apple extracts. Sample names are listed in Table 5.

Marzban et al. [25] classified apple cultivars as potentially low allergenic according to the Mal d 1 protein content, setting the threshold of allergenicity at 5 µg/g. In our study, we applied unsupervised integrative clustering (UIC) (Figure 2) taking into consideration not only the Mal d 1 content, but also the expression of *Mal d 1.06A* and the immunoreactivity datasets. We obtained three groups, potentially low (the right side of the chart), medium and highly allergenic cultivars (the left side of the chart) (Figure 2). Based on clustering (UIC) and the Marzban [25] criteria, we selected the following cultivars as potentially hypoallergenic: Kandil Sinap, Kosztela, Rumianka from Alma-Ata, Kantówka Gdańska, Reinette Coulon, and Gala (a new one).

In the present report, we assumed cv. Gala can be considered low allergenic for patients with Mal d 1 sensitization due to the low level of the Mal d 1 protein and encoded gene expression; even the immunoreactivity of all the patients’ sera with the fruit extract was low regardless of the apple fruit production method. Moreover, this genotype is easily accessible to consumers because of its popularity as a very good dessert variety.

According to Vlieg–Boerstra et al. [26], the prick-to-prick and skin prick test are not effective to determine the allergenicity of apple cultivars; therefore, we applied protein slot blotting with an immunoassay as a more reliable approach to assess fruit allergenicity. In the further study, we are going to extend the immunoreactivity analysis using sera of individuals sensitized not only to birch pollen and/or apples, but also to peaches.

The limitation of our study was the use of indigenous varieties that are not well-known, growing in small orchards or gene banks; however, old varieties could be an important source of low allergenic apples, as we have shown thereby.

To sum up, our approach to the issue of apple allergenicity made it possible to detect potentially low, medium and highly allergenic varieties. Up to date, only Topaz, Elise, and Santana varieties are considered to be well-tolerated by apple allergic patients [26,27]. Thanks to our study, we can expand the list by five old and one new cultivar that emerged as potentially hypoallergenic apples for patients sensitized to birch pollen exhibiting apple allergy. Our research showed that old varieties are a rich source of potentially hypoallergenic varieties.

This report extends the knowledge on the regulation of gene expression of apple allergens under the influence of genotype, farming method, or maturity of fruits, which may be useful in assessing the risk of allergic reaction occurrence after apple ingestion. We provide basis to consider the *Mal d 1.06A* isoform as more important in the determination of apple allergenicity than *Mal d 1.01*. The knowledge of the molecular mechanism of apple allergenicity and factors that modify reaction severity could facilitate medical counselling and improve care of patients with food allergies.

## 4. Materials and Methods

### 4.1. Plant Material

Apple fruits of 21 old apple cultivars (*Malus x domestica* Borkh.) (before the Green Rvolution) organically farmed were collected from the Arboretum and Institute of Physiography in Bolestraszyce, Poland (Table 1). Eight modern apple cultivars, organically and intensively grown, were collected from “BioGrim” company, Wojciechow, Poland. Three apple cultivars were collected from trees farmed integratively at the Institute of Horticulture in Brzezna, Poland. Fruits of the Santana cultivar (*Malus x domestica* Borkh. cv. Santana) were collected from a private orchard located near Bielsko-Biała, Poland. Apple skin and flesh of apple fruits were collected separately and stored at −80 °C. RNA purification followed sample lyophilization.

### 4.2. Human Samples

The four patients participating in the study were tested using the skin prick test and diagnosed with an allergy to birch pollen and other tree pollens. All participants provided informed consent for participation in this research program, which was approved by the Ethics Committee of Poznan University of Medical Sciences, Poland, document number 178/19.

### 4.3. RNA Extraction 

RNA extraction was performed according to the method used by Reid et al. [28] with some modifications. The tissue was ground in a prechilled mortar to a fine powder and then added to a prewarmed (65 °C) extraction buffer at a proportion of 0.15 g of tissue per 7.5 mL and shaken vigorously. The RNA extraction buffer contained 2% CTAB (Cetyltrimethylammonium bromide; Sigma-Aldrich, Saint Louis, MO, USA), 2 M NaCl, 300 mM Tris-HCl (pH = 8.0) (Sigma-Aldrich, Saint Louis, MO, USA), 0.25 mM EDTA (Sigma-Aldrich, Saint Louis, MO, USA), 0.05% spermidine (Sigma-Aldrich, Saint Louis, MO, USA), 1% polyvinylpyrrolidone (PVP) K-30 (soluble) (Sigma-Aldrich, Saint Louis, MO, USA), and 2% β-mercaptoethanol (Sigma-Aldrich, Saint Louis, MO, USA) added directly before use. The tubes were incubated in a water bath at 65 °C for 10–15 min and shaken for 10–15 s every 3 min. Subsequently, tubes were left to cool down to room temperature and equal volumes of chloroform/isoamyl alcohol (Sigma-Aldrich, Saint Louis, MO, USA) (24:1) were added to samples, centrifuged at 3500× *g* for 20 min at 4 °C. The aqueous layer was transferred to a new tube and centrifugated at 30,000× *g* for 15 min at 4 °C to remove any insoluble particles in the solution. For nucleic acid precipitation, 0.1 vol 3 M NaOAc (pH = 5.2 ± 0.2) (Sigma-Aldrich, Saint Louis, MO, USA) and 0.6 vol isopropanol (Sigma-Aldrich, Saint Louis, MO, USA) was added, mixed, and stored at −80 °C for 20 min. The samples were centrifuged at 3500× *g* for 30 min at 4 °C to collect the nucleic acid pellet. The supernatant was discarded and the pellet was dissolved in 1 mL TE (Tris(hydroxymethyl)aminomethane and ethylenediaminetetraacetic acid; Sigma-Aldrich, Saint Louis, MO, USA) buffer (pH 7.5) and transferred to a new 2 mL microcentrifuge tube. Messenger RNA was selectively precipitated by adding 0.3 vol 8 M LiCl (Sigma-Aldrich, Saint Louis, MO, USA) and stored for 25 h at 4 °C, then centrifuged at 20,000× *g* for 30 min at 4 °C. The messenger RNA pellet was then washed by adding 700 µL 70% ice-cold ethanol (Sigma-Aldrich, Saint Louis, MO, USA) and air-dried. The pellet was dissolved in 70–100 µL DEPC (Diethyl pyrocarbonate; Sigma-Aldrich, Saint Louis, MO, USA) water.

Quantity and quality of total mRNA were determined spectrophotometrically (Nanodrop Technologies LLC, Wilmington, DE, USA) by measuring OD260/230 and OD260/280. RNA integrity was assessed by inspection after agarose gel electrophoresis in the presence of SybrGreen Safe Stain (Ivitrogen, Carlsbad, CA, USA). Only mRNA samples meeting criteria 260/2880 > 1.9 and 260/280 ≥ 2.0 were taken to cDNA synthesis.

### 4.4. cDNA Synthesis

Reverse transcription was performed with a TranScriba Kit (A&A Biotechnology, Gdansk, Poland) according to the manufacturer’s instructions using oligo(dT) primers and 250 ng total mRNA. Controls with no transcriptase were used to assess the potential contamination of genomic DNA. The cDNA concentration was assessed spectrophotometrically (Nanodrop Technologies LLC, Wilmington, DE, USA).

### 4.5. Gene Expression

Gene expression was determined by real-time PCR using a thermal cycler (Lightcycler-96; Roche Molecular Systems, Pleasanton, CA, USA). The oligonucleotide primer sequences used for real-time PCR (Table 6) were adopted from the literature [7,15,29,30]. The amplification efficiency was evaluated for every primer pair using regression and the slope according to the following equation: 10^(–1/slope)^ [31]. Relative expression was normalized to the geometric mean of reference gene expression levels (actin and ubiquitin genes) and expressed as arbitrary units (AU).

### 4.6. Extraction of Apple Proteins

Frozen apple flesh in the amount of 0.5 g was shaken with 5 mL of 10 mM PBS buffer (Sigma-Aldrich, Saint Louis, MO, USA), pH = 5.4, for 1 h at room temperature followed by centrifugation at 4 °C for 30 min at maximum speed. Every sample was extracted in duplicate.

For further analysis, 2 mL of a supernatant were taken and centrifuged at 15,000 rpm for 10 min, and then the supernatant was transferred to a new tube. Supernatants we stored at −80 °C until used in the following procedure.

### 4.7. Protein Slot Blotting

Blotting was performed using a Slot Blotter (Geneflow, Lichfield, England). In the procedure, 150 µL of the extracted protein solution were taken. Before blotting, a PVDF membrane (Merck, Darmstadt, Germany) (4.5 cm × 4.4 cm) was activated by immersion in methanol and soaked with a transfer buffer (12 mM Tris, 96 mM glycine, and 20% methanol) (Bio-Rad, Hercules, CA, USA) for 5 min. The activated membrane was placed between the tissue paper soaked with the transfer buffer beforehand in the apparatus. After immobilization of apple proteins, the membrane was incubated with the blocking solution containing 1% BSA (Bio-Rad, Hercules, CA, USA) in a TBS buffer (20 mM Tris, pH 7.5, 500 mM NaCl) (Bio-Rad, Hercules, CA, USA) for 45 min with gentle shaking at room temperature. Blocking was followed by washing three times for 5 min in a TBS–Tween buffer (Bio-Rad, Hercules, CA, USA), pH 7.4.

For the immunoassay, human sera (containing IgE primary antibodies) were 10–100× diluted with a 1% BSA–TBS buffer. Membranes were then incubated with a diluted solution of sera for 45 min and washed five times with a TBS–Tween buffer. For immunodetection, 1:1000 diluted secondary polyclonal goat antihuman IgE antibody conjugated with alkaline phosphatase (Invitrogen) was used for 45 min. Incubation was followed by five washes with TBS–Tween, pH 7.4. Blots were then stained with 5 mg BCIP (5-bromo-4-chloro-3′-indolyphosphate p-toluidine salt; Bio-Rad, Hercules, CA, USA) and 10 mg NBT (nitro-blue tetrazolium chloride; Bio-Rad, Hercules, CA, USA) for 20 min. The reaction was stopped by washing with water. After staining, membranes were air-dried and photographed. 

### 4.8. ELISA

Microtiter plates were activated with 200 μL carbonate/bicarbonate buffer, pH = 9.6, at 37 °C for 30 min. After activation, microtiter plates were coated with 100 μL of apple protein extracts at 37 °C for 120 min. As standards, rMal d1 (Mal d1.0108, Biomay, Wien, Austria) (1000 ng/mL–7.81 ng/mL) and rBet v1-A (Cusabio Technology LLC, Houston, TX, USA) (138 ng/mL–1.08 ng/mL) allergen proteins were used. Free binding sites were blocked with 1% BSA in a TBS buffer, pH = 7.4, for 45 min. Specific Bet v 1 rabbit IgG polyclonal antibody (100 μL; diluted 1:2000) was added and the plate was incubated at 37 °C for 90 min. Immunocomplexes of Mal d 1 and IgG were detected by monoclonal anti-rabbit IgG peroxidase antibody (1:400,000; SIGMA A1949; Sigma-Aldrich, Saint Louis, MO, USA) and incubated at 37 °C for 60 min without mixing. Color reaction was developed after the addition of 200 μL o-phenylenediamine dihydrochloride (OPD;Sigma-Aldrich, Saint Louis, MO, USA) and incubated for 30 min. The reaction was stopped by adding 50 μL 3M H2SO4 (Sigma-Aldrich, Saint Louis, MO, USA). The absorbance was measured at 492 nm using a spectrophotometer. 

### 4.9. Statistical Analysis

The data are expressed as the means or medians and were analyzed using the statistical program Statistica v.13 (StatSoft, Krakow, Poland). *P*-values < 0.05 were considered statistically significant. The homogeneity of variance for all the data was assumed using the Levene’s test. The one-way analysis of variance (ANOVA) was used for assessing differences between groups. Differences between the average values of the old and new apple cultivars were analyzed using the unpaired *t*-test. For evaluating correlations, the Pearson’s procedure (normally distributed data) was used, in which the *p*-value was considered to be statistically significant at < 0.05. Differences between groups of differentially cultivated apple trees were assessed using the Tukey’s test.

Unsupervised, integrative clustering. The integration of gene expression (mRNA), immunoreactivity, and protein expression was performed using the R/Bioconductor package moCluster [32]. In brief, this method relies on multiblock multivariate analysis that defines a set of latent variables representing joint patterns across input datasets, which is further passed to a hierarchical clustering algorithm (Euclidean distance measurement and Ward linkage) in order to discover joint clusters. The decision on an optimal number of clusters was made based on the gap statistic [33].

Hierarchical classification on principal components (HCPC) using gene expression data using the FactoMineR R package was performed.

## Figures and Tables

**Table 1:** Mean expression of genes encoding four main apple allergens in arbitrary units (AU).

		Old Cultivars	New Cultivars			
Tissue	Gene	Organic	All New	Organic	Intensive	Integrated
Flesh	*Mal d 1.06A*	3.43	2.90	2.74	3.04	2.99
	*Mal d 1.01*	3.61	3.37	3.44 (g)	3.38	2.95 (g)
	*Mal d 2.01*	4.12 (b,d,p)	3.69 (d)	3.35 (a,b,c)	4.01 (c)	3.66 (a)
	*Mal d 3.01*	3.05 (e)	2.88	3.13	2.77	2.61
	*Mal d 4.01*	3.30	3.23	3.29	3.2	2.96
Skin	*Mal d 1.06A*	3.90 (h)	3.33	3.40	3.51	2.93
	*Mal d 1.01*	4.32 (h)	3.78	3.91	4.01	2.9
	*Mal d 2.01*	3.56 (p)	3.31	3.05	3.67	2.96
	*Mal d 3.01*	4.97 (e)	4.54	4.94	4.96	3.02
	*Mal d 4.01*	2.81	2.89	2.85	2.93	2.78
Average	*Mal d 1.06A*	3.67 (f,l)	3.12 (l)	3.07	3.28	2.96
	*Mal d 1.01*	3.97 (f,n)	3.57 (n)	3.68 (m)	3.70	2.93 (m)
	*Mal d 2.01*	3.84 (j,k)	3.50 (j)	3.20 (i,k)	3.84 (i)	3.31
	*Mal d 3.01*	3.99	3.71	4.04 (r)	3.86 (r)	2.81 (r)
	*Mal d 4.01*	3.05	3.06	3.07	3.10	2.87

a–n,p,r—the same letter indicates groups whose means differ significantly at *p* < 0.05.

**Table 2:** Mal d 1 protein content in apple flesh.

	Apple Cultivars	Mal d 1 Content (µg/g FW ^1^)
Old organic farming	Kantówka Gdańska	0.3
	Kosztela	0.6
	Antonovka Usual	1.8
	Sztetyna	1.9
	Rumianka from Alma-Ata	2
	Reinette Coulon	2.5
	Żeleźniak	3.5
	Kandil Sinap	4.6
	Bukówka	7.3
	Emperor Alexander Apple	7.6
	Jonathan	7.7
	Antonovka One and a Half Pound	7.7
	Grochówka	8.2
	Oberland Raspberry Apple	10
	Winter Banana	12.9
	Jakub Lebel	17.5
	Gloria Mundi	20.9
	Gray French Reinette	23.5
	Reinette de Canada	28.8
	Berner Rose	37.7
	**Median**	**7.65**
New organic farming	Gala	1.3
	Golden Delicious	1.8
	Jonagored	5.8
	Idared	6
	Santana	8.5
	Trinity I (x Gold Millennium)	12.7
	Gold Millennium	13.2
	Trinity II (x Ligol)	15
	**Median**	**7.25**
New intensive farming	Gold Millennium	2.3
	Gala	2.4
	Idared	2.4
	Golden Delicious	5.8
	Jonagored	9.5
	Trinity	13.6
	**Median**	**4.1**
**Median in new**		**5.9**

^1^ FW—fresh weight.

**Table 3:** Correlation between the mean expression and fruit maturity.

	*Mal d 1.06A*	*Mal d 1.01*	*Mal d 2.01*	*Mal d 3.01*	*Mal d 4.01*
Pearson’s r	0.5211	0.6129	0.3461	0.4012	0.6175
*p*-value	0.038	0.012	0.189	0.123	0.011

**Table 4:** Correlation between immunoreactivity of patients’ sera, *Mal d 1.06A* expression, and Mal d 1 content.

		Mal d1 (µg/g FW ^1^)	Serum 1	Serum 2	Serum 3	Serum 4
Expression of *Mal d 1.06A*	Pearson’s r coeff.	0.38	0.4	0.34	0.4	0.37
	*p*-value	0.036	0.027	ns	0.028	0.046
Mal d1 (µg/g FW)	Pearson’s r coeff.	-	0.24	0.25	0.22	0.39
	*p*-value	-	ns	ns	ns	0.031

^1^ FW—fresh weight; ns—not significant.

**Table 5:** Characteristics of apple fruit samples used in the study.

Type of Varieties	Sample Name	Cultivar Name	Cultivation Method	Sample Origin
Old	X_15	Rumianka from Alma-Ata	organic	Bolestraszyce
	X_17	Sztetyna	organic	Bolestraszyce
	X_19	Gloria Mundi	organic	Bolestraszyce
	X_21	Kosztela	organic	Bolestraszyce
	X_23	Reinette Coulon	organic	Bolestraszyce
	X_25	Emperor Alexander Apple	organic	Bolestraszyce
	X_27	Kantówka Gdańska (Danzinger Kantapfel)	organic	Bolestraszyce
	X_29	Żeleźniak (Rother Eiserapfel)	organic	Bolestraszyce
	X_31	Jonathan	organic	Bolestraszyce
	X_33	Reinette de Canada	organic	Bolestraszyce
	X_35	Oberland Raspberry Apple (Callville d’Automne Raye)	organic	Bolestraszyce
	X_37	Bukówka	organic	Bolestraszyce
	X_39	Jakub Lebel	organic	Bolestraszyce
	X_41	Winter Banana	organic	Bolestraszyce
	X_43	Kandil Sinap	organic	Bolestraszyce
	X_45	Parker’s Pippin	organic	Bolestraszyce
	X_47	Gray French Reinette	organic	Bolestraszyce
	X_51	Grochówka (Grosser Bohnapfel)	organic	Bolestraszyce
	X_53	Berner Rose	organic	Bolestraszyce
	X_103	Antonovka Usual	organic	Bolestraszyce
	X_105	Antonovka One and a Half Pound	organic	Bolestraszyce
Modern	X_80B	Jonagored	organic	Wojciechow
	X_81B	Jonagored	intensive	Wojciechow
	X_82A	Gold Millennium	organic	Wojciechow
	X_83A	Gold Millennium	intensive	Wojciechow
	X_84B	Gala	organic	Wojciechow
	X_85A	Gala	intensive	Wojciechow
	X_86A	Idared	organic	Wojciechow
	X_87A	Idared	intensive	Wojciechow
	X_88A	Golden Delicious	organic	Wojciechow
	X_89A	Golden Delicious	intensive	Wojciechow
	X_91A	Trinity	intensive	Wojciechow
	X_93A	Trinity I (× Gold Millennium)	organic	Wojciechow
	X_94A	Trinity II (× Ligol)	organic	Wojciechow
	X_96A	Idared	integrated	Brzezna
	X_97A	Gala	integrated	Brzezna
	X_99A	Golden Delicious	integrated	Brzezna
	X_108	Golden Delicious 2	organic	Wojciechow
	X_109	Golden Delicious 2	intensive	Wojciechow
	X_110	Idared 2	intensive	Wojciechow
	X_111A	Idared 2	organic	Wojciechow
	X_112A	Santana	organic	Bielsko-Biała
	X_113B	Golden Delicious 2	integrated	Brzezna

**Table 6:** Real-time PCR primers sets.

Gene	Primers	Sequence (5′–3′)	Access No.
*Mal d 1.01*	Md1-1.01F	AAGCTGAAATCCTTGAAGGAA	AJ417551
	Md1-1.01R	GTGCTCTTCCTTGATTTCAATG	
*Mal d 1.06A*	Mal d 1.06AF	TTGTTGCCAGATGGATGGTC	AY428580
	Mal d 1.06AR	TTGATGCTGACAATCTCATT	
*Mal d 2.01*	Mal d 2.01 F	GTGTGCCCGGCTCCACTT	AJ243427
	Mal d 2.01 R	TTCGAATCACCAAACGCAAG	
*Mal d 3.01*	Mal d3.01F	GTGACCAGCAGCCTTGCG	AF221502
	Mal d 3.01R	TTCAGGCAGTTGCAAGCAGT	
*Mal d 4.01*	Mal d 4.01F	GCTCTGGTGGCGTAACTGTG	AF129426
	Mal d 4.01R	CCTGGAGTCAAAGGCTCCTC	
*MdUBI*	UBI-F	TTGATCTTTGCTGGGAAACAG	CN491263
	UBI-R	CACCACCATCATTCAACACC	
*MdActin*	Actin-F	TGACCGAATGAGCAAGGAAATTACT	CN938023
	Actin-R	TACTCAGCTTTGGCAATCCACATC	

## Data Availability

Supporting data are in the Supplementary Data section.

## Supplementary Materials

The following are available online at https://www.mdpi.com/article/10.3390/ijms22073527/s1, Figure S1. *Mal d 1.06A* expression in fruits of old and new varieties organically cultivated; Figure S2. *Mal d 1.06A* expression in fruits of the selected new cultivars differentially farmed; Figure S3. *Mal d 1.01* expression in fruits of old and new varieties organically cultivated; Figure S4. *Mal d 1.01* expression in fruits of the selected new cultivars differentially farmed; Figure S5. *Mal d 2.01* expression in fruits of old and new varieties organically cultivated; Figure S6. *Mal d 2.01* expression in fruits of the selected new cultivars differentially farmed; Figure S7. *Mal d 3.01* expression in fruits of old and new varieties organically cultivated; Figure S8. Mal d 3.01 expression in fruits of the selected new cultivars differentially farmed; Figure S9. *Mal d 4.01* expression in fruits of old and new varieties organically cultivated; Figure S10. *Mal d 4.01* expression in fruits of the selected new cultivars differentially farmed.
