# Supplementary material for: Molecular and Immunological Identification of Low Allergenic Fruits among Old and New Apple Varieties
Source: Int J Mol Sci. 2021 Mar 29;22(7):3527. doi: 10.3390/ijms22073527 (PMC8036863; doi:10.3390/ijms22073527)

Figure S1. *Mal d 1.06A* expression in fruits of old and new varieties organically cultivated

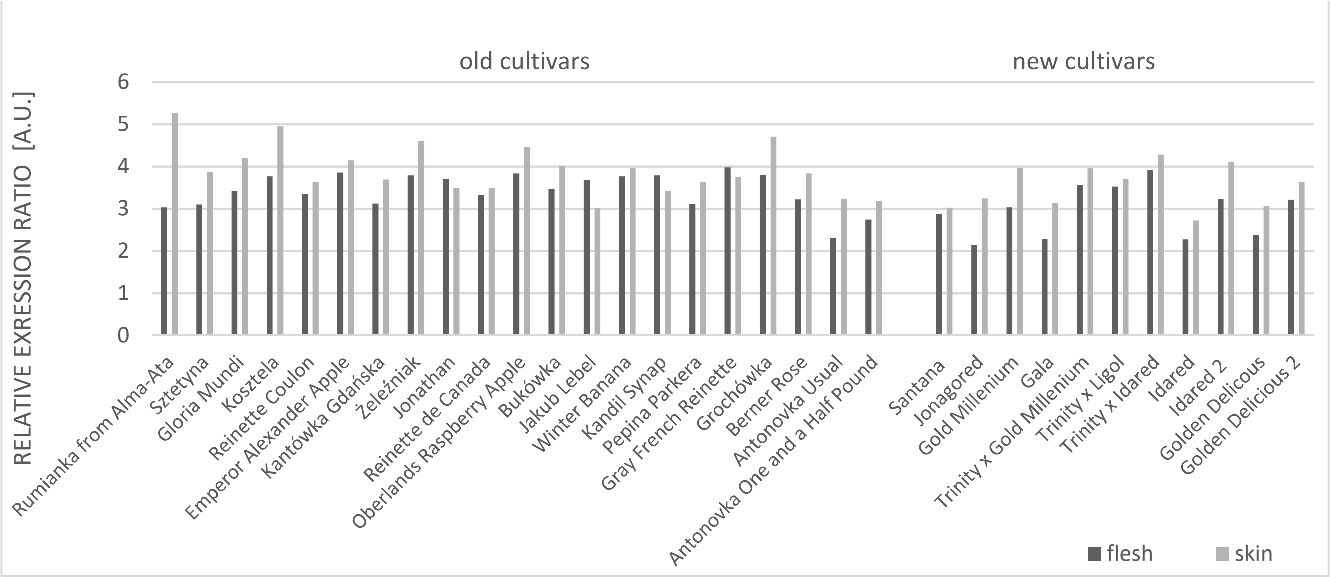

The number 2 followed the names of Idared and Golden Delicious indicates higher maturity status

Figure S2. *Mal d 1.06A* expression in fruits of the selected new cultivars differentially farmed

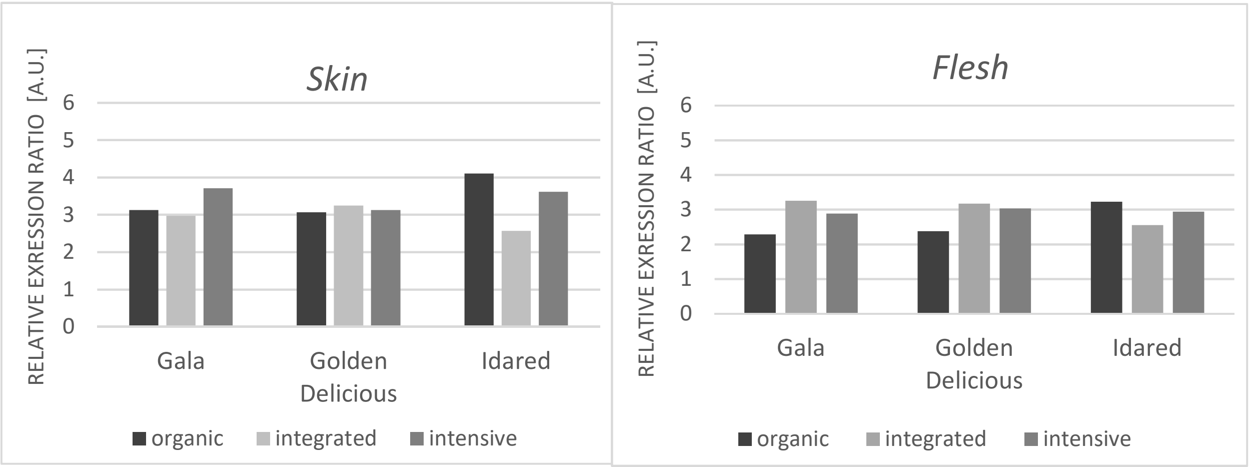

Figure S3. *Mal d 1.01* expression in fruits of old and new varieties organically cultivated

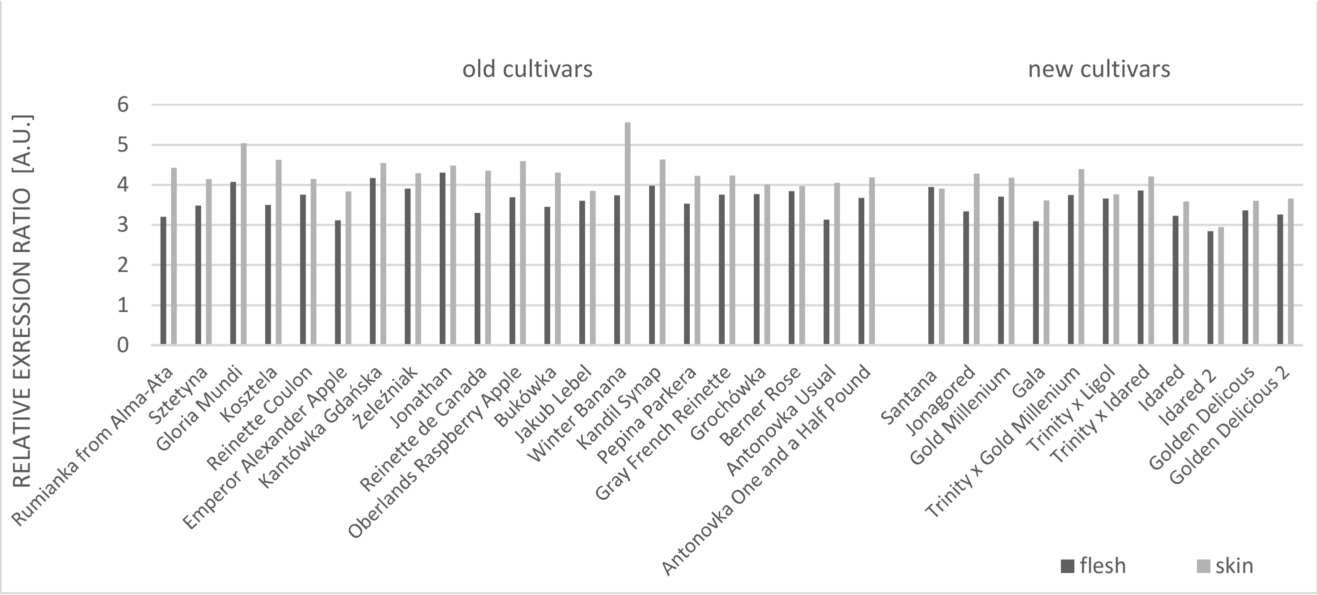

The number 2 followed the names of Idared and Golden Delicious indicates higher maturity status

Figure S4. *Mal d 1.01* expression in fruits of the selected new cultivars differentially farmed

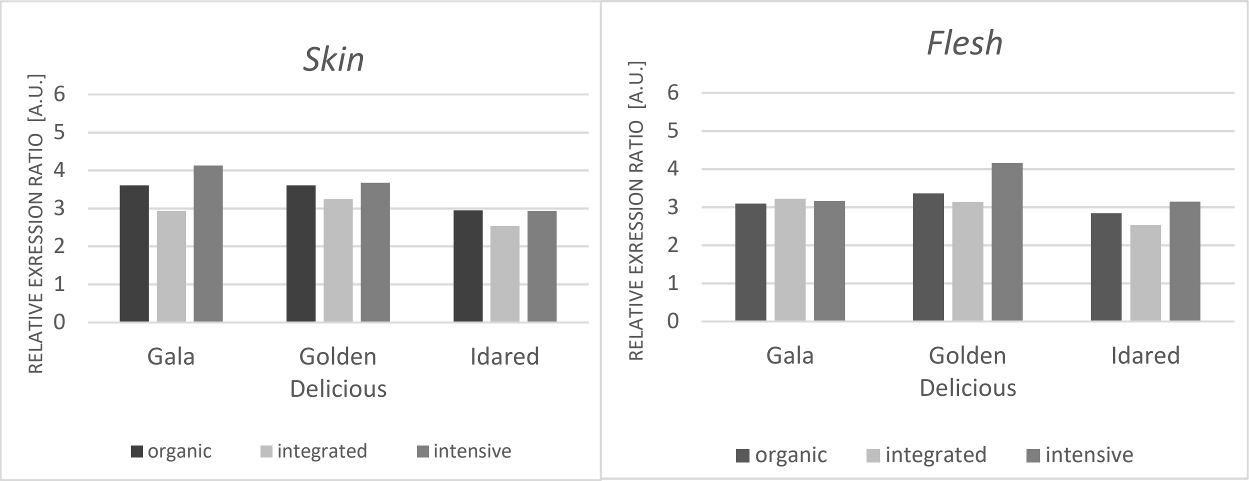

Figure S5. *Mal d 2.01* expression in fruits of old and new varieties organically cultivated

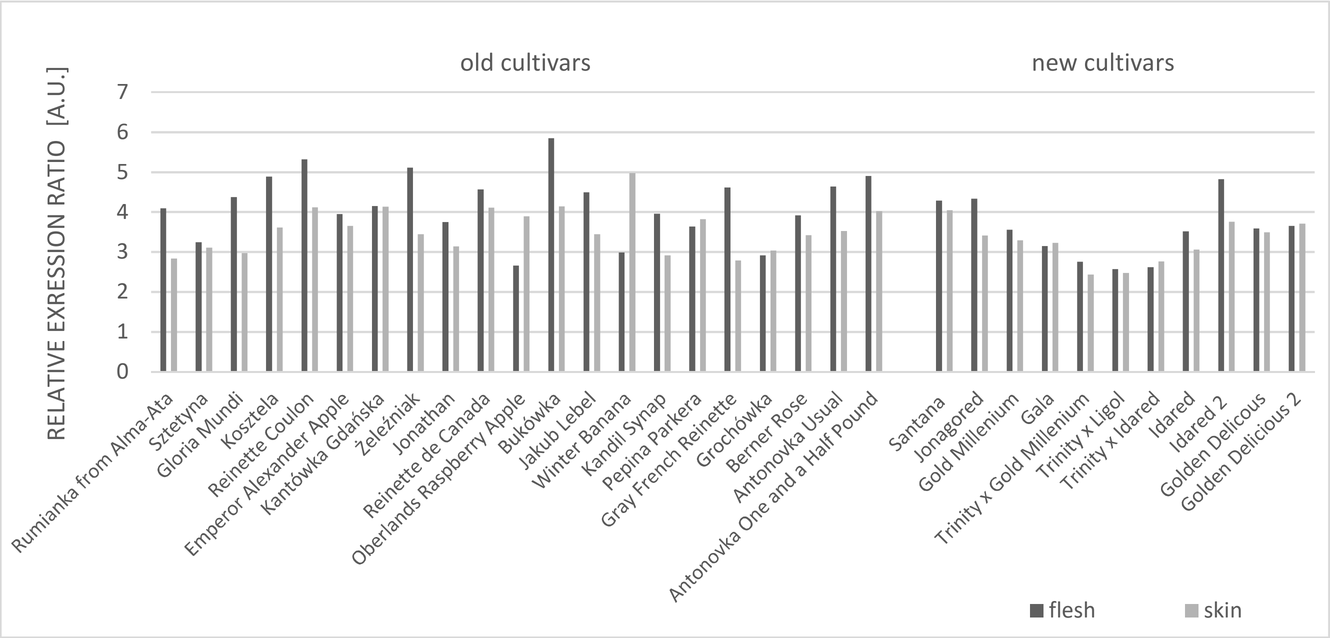

The number 2 followed the names of Idared and Golden Delicious indicates higher maturity status

Figure S6. *Mal d 2.01* expression in fruits of the selected new cultivars differentially farmed

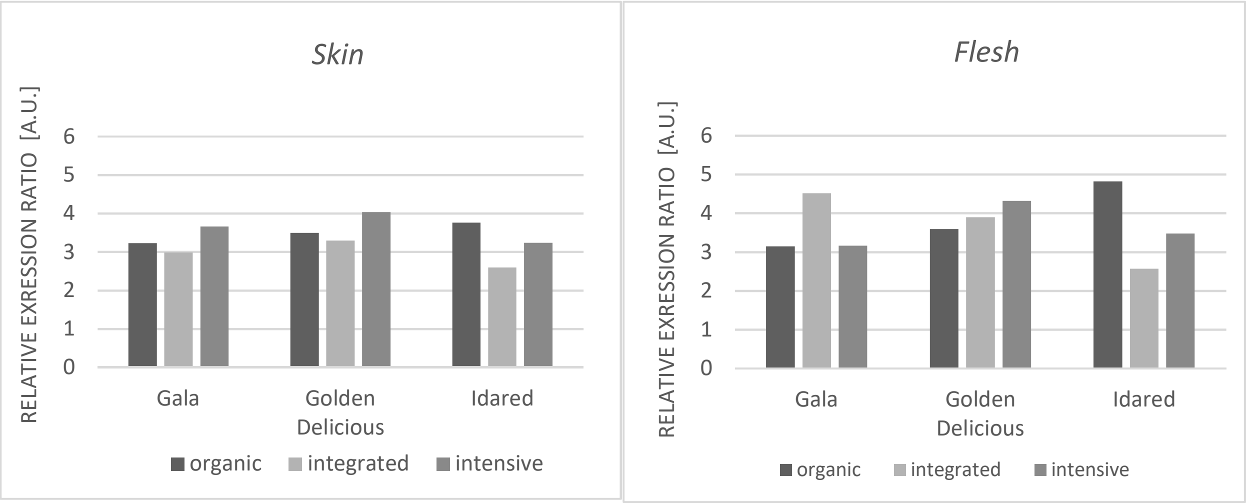

Figure S7. *Mal d 3.01* expression in fruits of old and new varieties organically cultivated

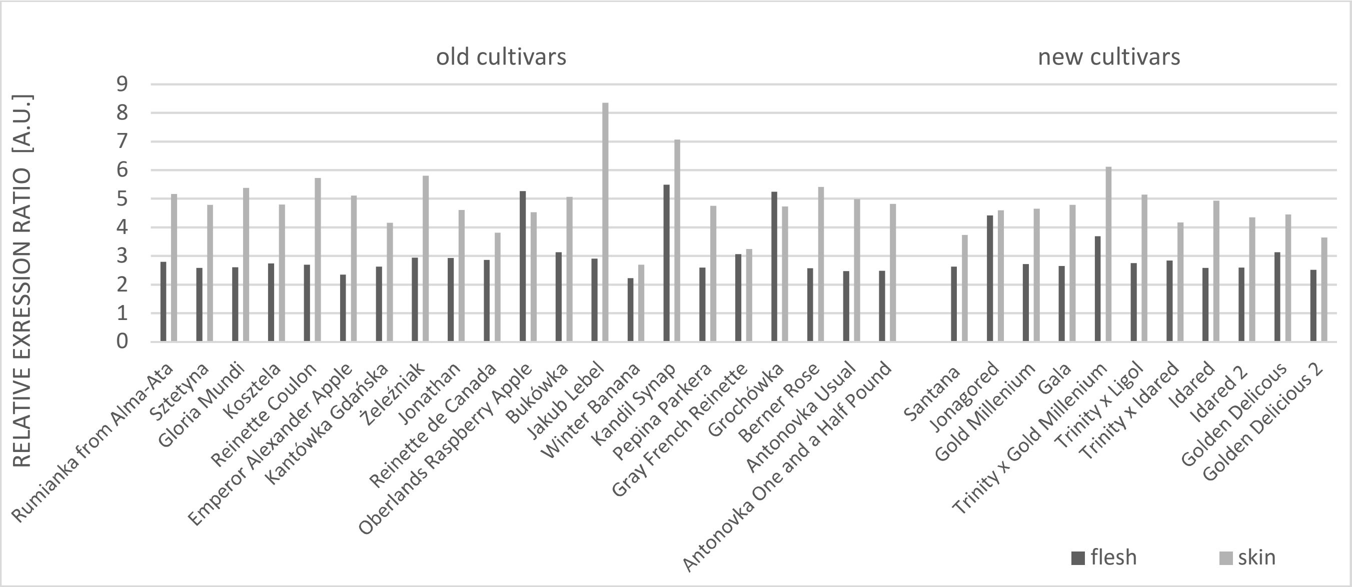

The number 2 followed the names of Idared and Golden Delicious indicates higher maturity status

Figure S8. *Mal d 3.01* expression in fruits of the selected new cultivars differentially farmed

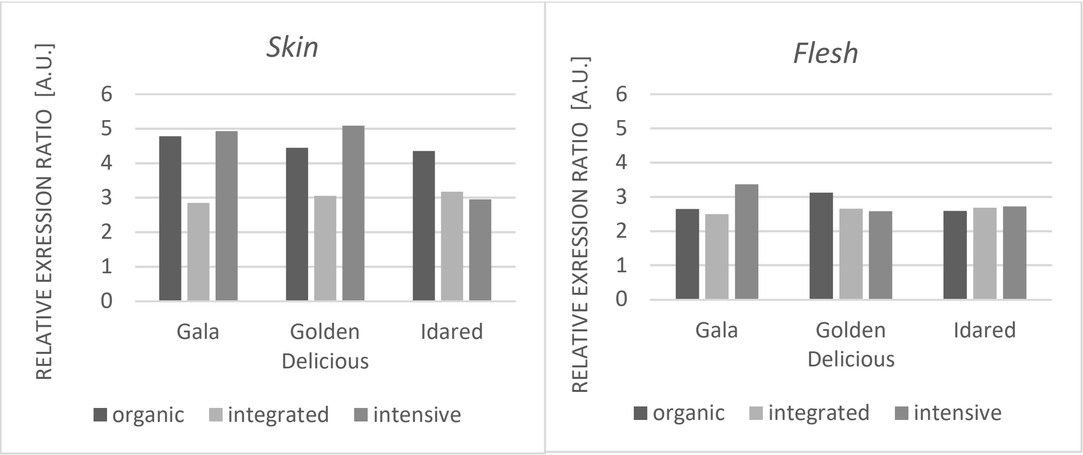

Figure S9. *Mal d 4.01* expression in fruits of old and new varieties organically cultivated

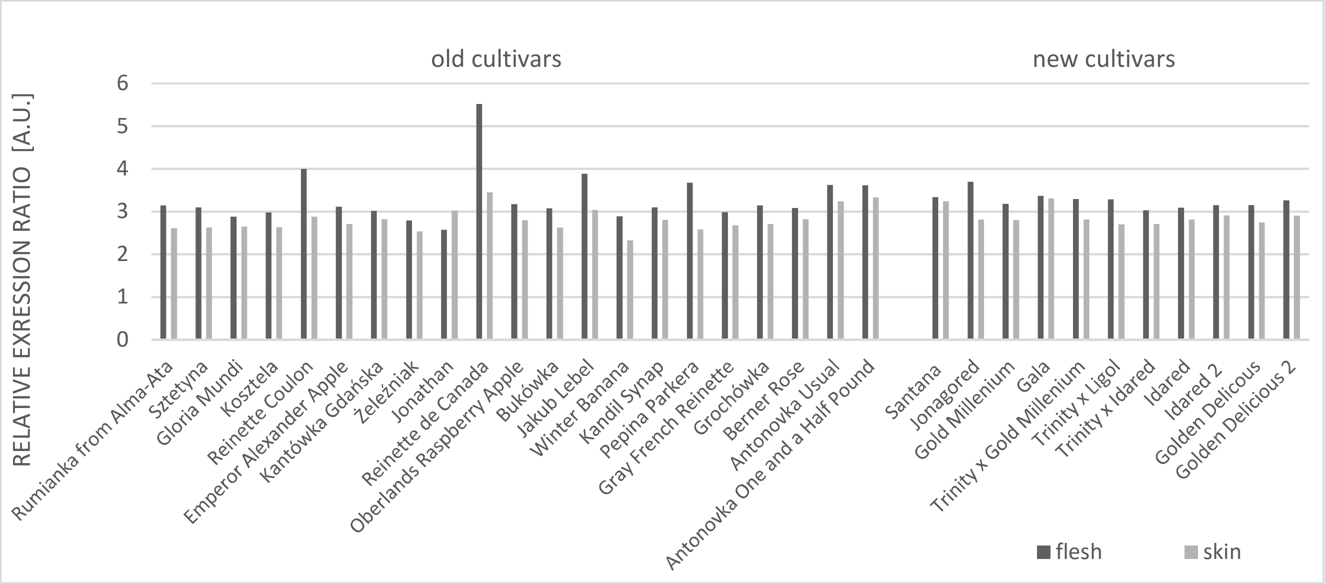

The number 2 followed the names of Idared and Golden Delicious indicates higher maturity status

Figure S10. *Mal d 4.01* expression in fruits of the selected new cultivars differentially farmed

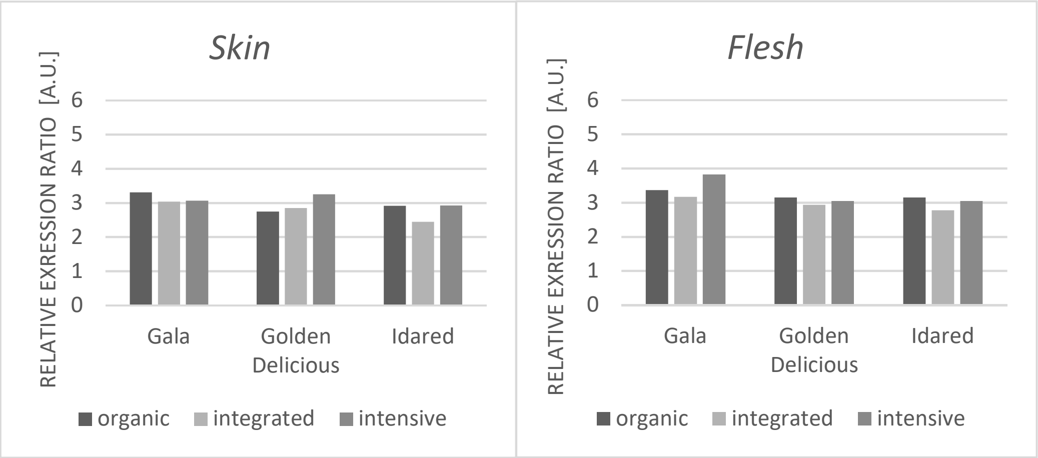

Supplement: Supplementary file 1 [file ijms-22-03527-s001.pdf]
